# Supplementary material for: Androgens predispose males to monocyte-mediated immunopathology by inducing the expression of leukocyte recruitment factor CXCL1
Source: Nat Commun. 2020 Jul 10;11:3459. doi: 10.1038/s41467-020-17260-y (PMC7351718; doi:10.1038/s41467-020-17260-y)
Supplement: Supplementary file 1 — Supplementary Information [file 41467_2020_17260_MOESM1_ESM.pdf]

Supplementary Information

Androgens predispose males to monocyte-mediated immunopathology by inducing the expression of leukocyte recruitment factor CXCL1

Sellau *et al*.

This PDF file includes
Supplementary Figures 1 to 4

Supplementary Figure 1

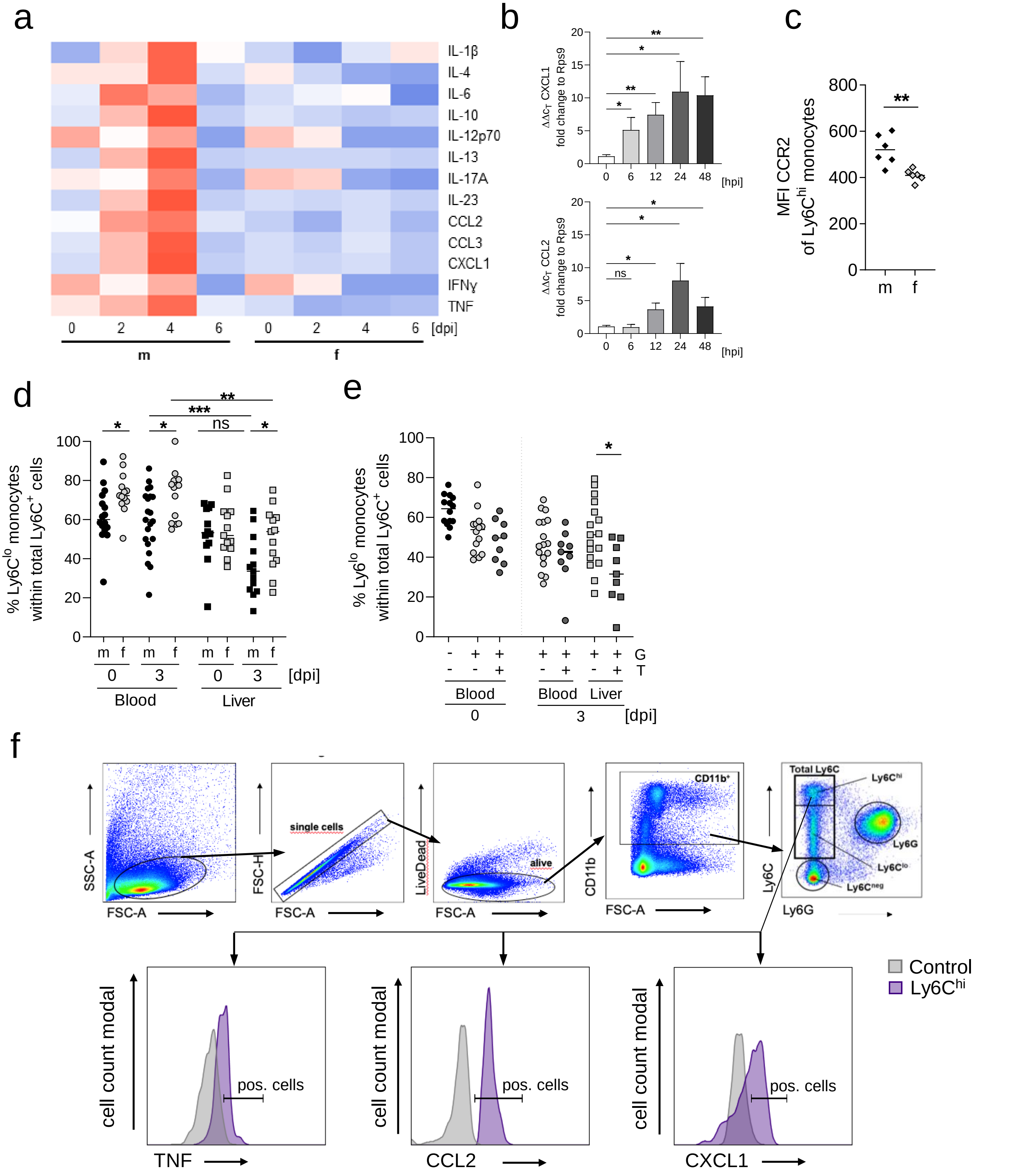

Supplementary Figure 1: Cytokines, Ly6C^{lo} monocytes, and the effect of testosterone treatment during *E. histolytica* infection. (a) Multiplex analysis of cytokines in the plasma of male and female mice at different time points following intrahepatic infection with *E. histolytica,* plotted as a heatmap (pooled data of two independent experiments). **(b)** Expression of mRNA encoding CCL2 and CXCL1 in the liver during early infection (CXCL1: $n_{m\ hpi}$=5; $n_{m\ 6,\ 12,\ 24,\ 48\ hpi}$=4; : $n_{m\ 0\ hpi}$=5; $n_{m\ 6,\ 12,\ 24,\ 48\ hpi}$=4; Depicted are the means ± SEM). **(c)** MFI of CCR2 of Ly6C^{hi} monocytes in the bone marrow of naïve male and female mice (n=6; data shown are one representative out of two independent experiments; Depicted are the means). **(d)** Frequency of Ly6C^{lo} monocytes within total liver-derived Ly6C^{+} monocytes within the CD11b^{+} monocyte population from the blood and the liver in naïve and *E. histolytica*-infected male and female mice (Day 3 p.i.) blood: $n_{m/f\ d0}$=16/13; $n_{m/f\ d3}$=21/14; liver: $n_{m/f\ d0}$=12/13; $n_{m/f\ d3}$=13/13; pooled data of four independent experiments; Depicted are the means). **(e)** Percentage of Ly6C^{lo} monocytes in the blood and liver of naïve or *E. histolytica*-infected G and T male mice (blood: $n_{wt/G/T\ d0}$=14/14/9; $n_{G/T\ d3}$=17/9; liver: $n_{G/T\ d3}$=17/9;; pooled data of two independent experiments; Depicted are the means ± SEM). **(f)** Gating strategy to identify Ly6C^{hi} and Ly6C^{lo} monocytes, as well as expression of TNF, CCL2 and CXCL1 (Fig.1g-j,l; 2a-d; 7b-f; S1 c-e). P-values were calculated using two-tailed grouped analysis: Student's t-test (b, c) or the Mann-Whitney test (d, e), *P < 0.05; **P < 0.01; ***P < 0.001. Source data are provided as a Source Data file.

Supplementary Figure 2

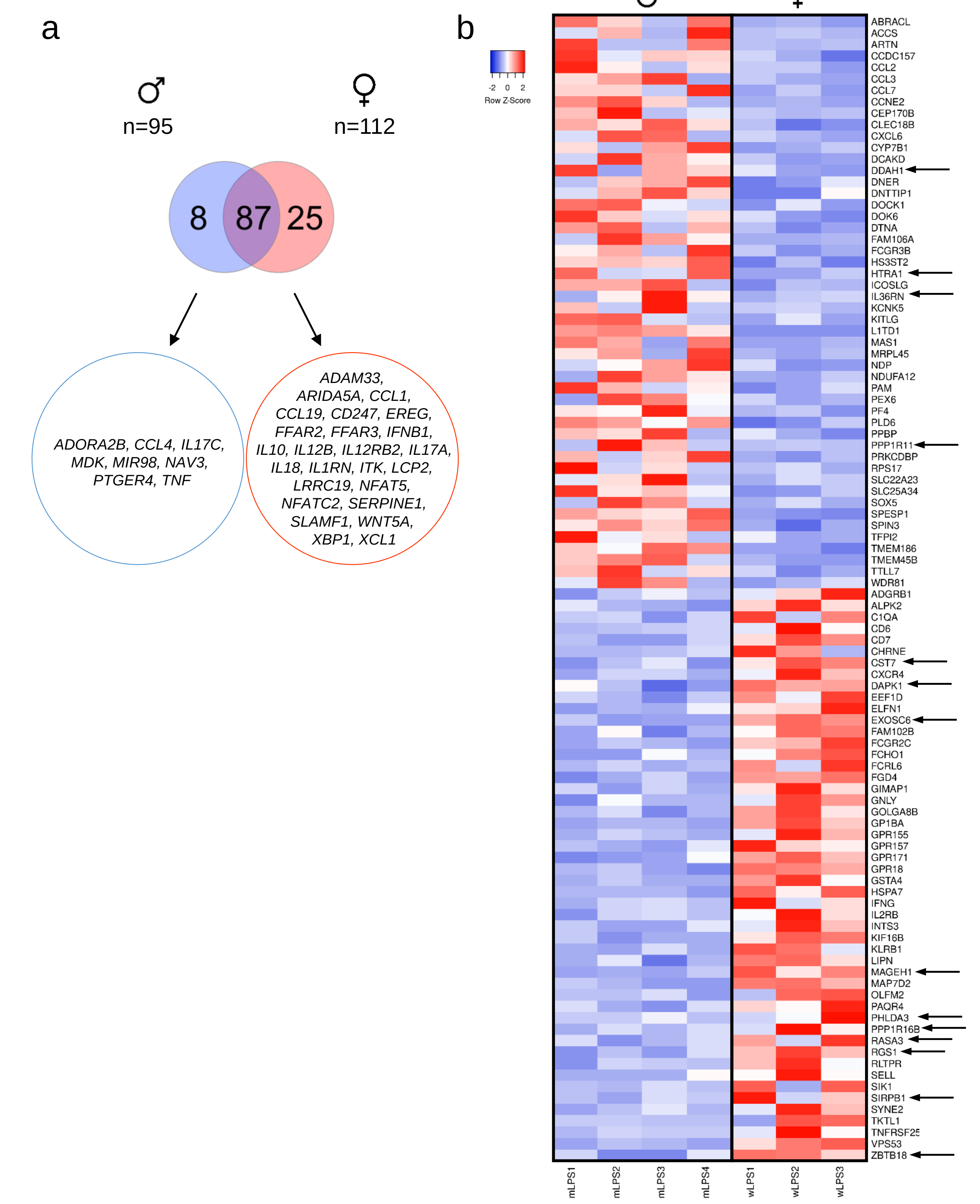

Supplementary Figure 2: Transcriptome analysis of CD14⁺ monocytes from men and women. PBMCs from healthy male (n=4) and female (n=3) donors were stimulated with LPS, and CD14⁺ monocytes were purified using MACS. RNA was isolated, transcribed into cDNA, and subjected to RNA Seq analysis. Genes falling into the following criteria were used for further analysis: log2FoldChange > 2 and a padj value < 0.05. **(a)** Venn diagram showing differentially regulated genes from men and women falling within the GO term cytokine production (GO:0001816). Differentially expressed genes are listed below. The heatmap displays the 50 most differentially expressed genes between male- and female-derived monocytes following LPS stimulation **(b)**. Genes playing a role in immune regulation are highlighted and listed in **Supplementary Table S1**.

Supplementary Figure 3

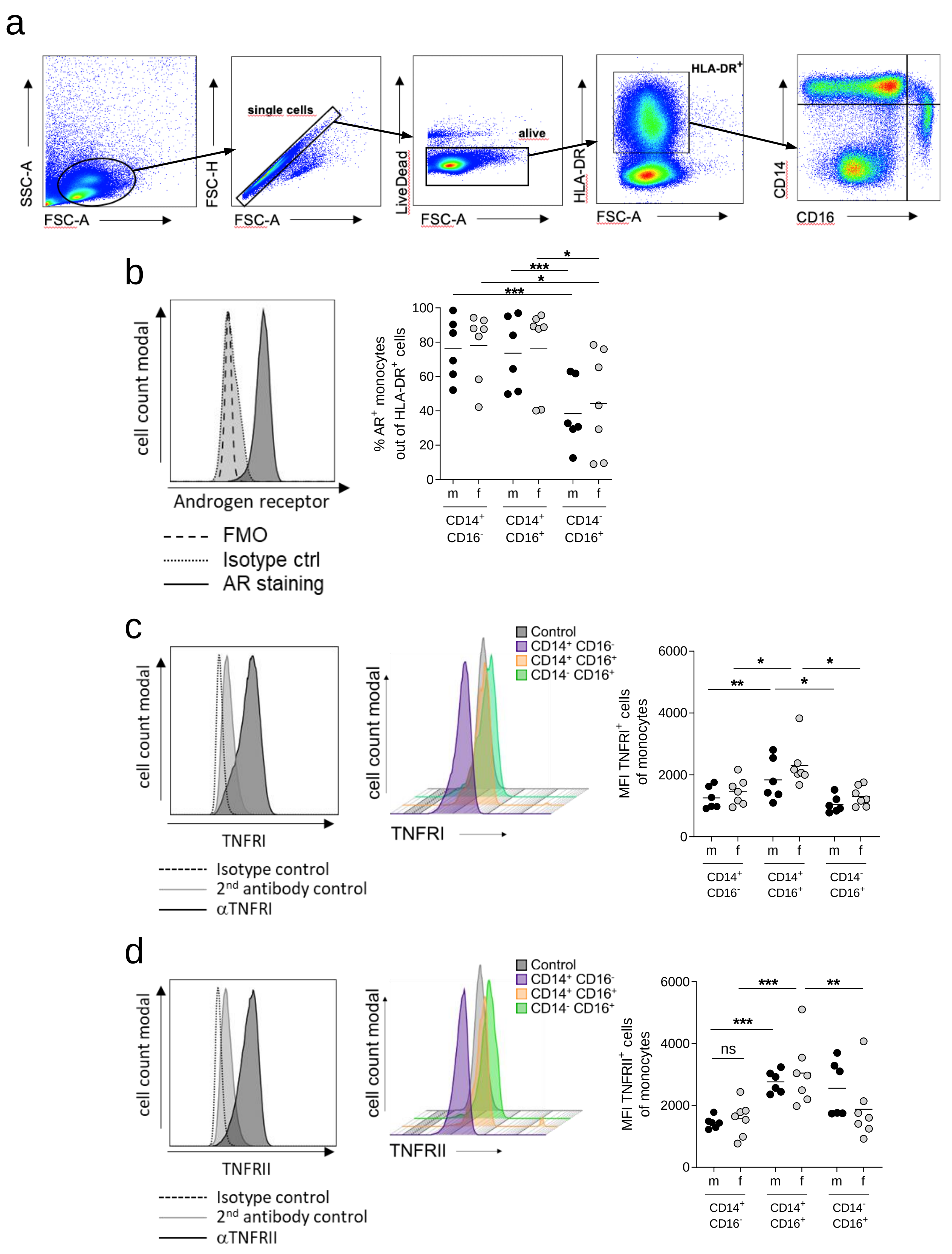

Supplementary Figure 3: Expression of the and TNFRI/II by peripheral human monocyte subpopulations. (a) Gating strategy to identify different human monocyte subsets (Fig. 3a-e; S3b-d). **(b)** Histogram showing androgen receptor (AR) staining and expression patterns among classical, intermediate, and non-classical monocytes ($n_{m/f}$=7/6; pooled data from samples collected over a time frame of nine months; Depicted are the means). Histogram of TNFRI **(c)** and TNFRII **(d)** staining and expression patterns among classical, intermediate, and non-classical monocytes ($n_{m/f}$=7/6; pooled data from samples collected over a time frame of nine months; Depicted are the means). P-values were calculated using two-tailed grouped analysis: Mann-Whitney test (a,b,c), *P < 0.05; **P < 0.01; ***P < 0.001. Source data are provided as a Source Data file.

Supplementary Figure 4

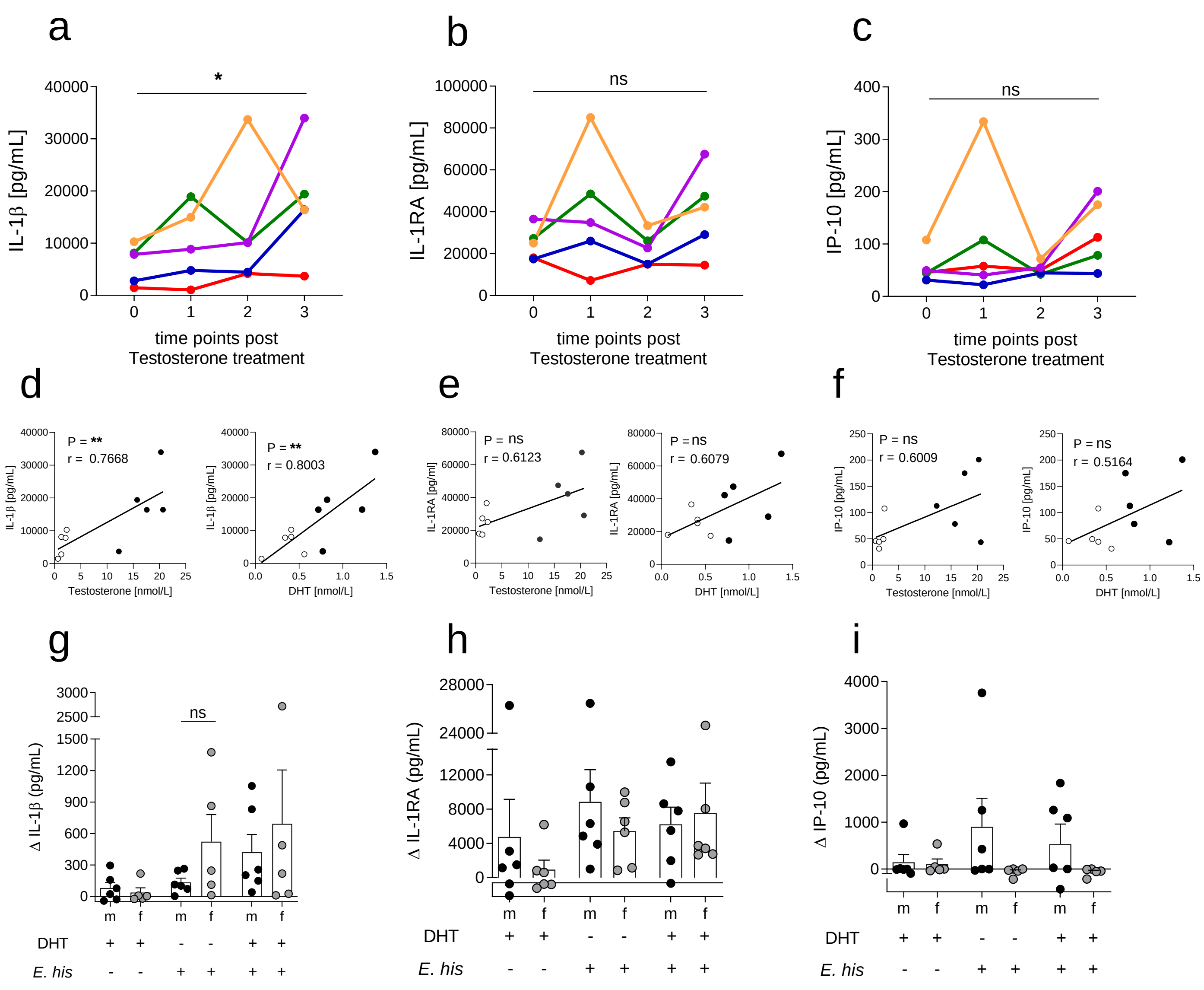

Supplementary Figure 4: Effect of androgens on immune responses. Cytokine production by PBMCs isolated from five women undergoing gender transformation through hormone replacement therapy. Women received testosterone and PBMCs were collected at different times from initiation of hormone treatment (0 (Day -9–0); 1 (Day 39–42); 2 (Day 112–126); 3 (Day 201–219)) and stimulated with LPS (0.1 µg/mL) for 17 h. **(a)** IL-1β, **(b)** IL-1RA, and **(c)** IP-10 concentrations in the supernatant were measured using a Multiplex Cytokine Assay. Each donor of the cohort is depicted in an individual color (n=5). Correlation between testosterone and DHT plasma levels in transgender men and **(d)** IL-1β **(e)** IL-1RA, and **(h)** IP-10 expression before transformation and at time point 3 post-transformation. Treatment of isolated peripheral**,** MACS-purified monocytes from healthy men and women with *E. histolytica* lysate (0.1 mg/mL) either alone or in the presence of DHT (10 nM) for 24 h. **(g)** IL-1β, **(h)** IL-1RA, and **(i)** IP-10 concentrations in the supernatant were determined using a Multiplex Cytokine Assay (n=5–6; pooled data from three independent experiments; Depicted are the means ± SEM; To normalize the baseline of the donors, values for unstimulated controls were subtracted from the stimulated samples). (a-c) P-values were calculated using two-tailed paired analysis student's t-test (*P < 0.05) or (d-f) two-tailed Pearson correlation coefficient (r; *P < 0.05, **P < 0.01) with a simple linear regression. Source data are provided as a Source Data file.

Supplementary Table 1: Differentially expressed regulatory genes of male and female-derived LPS-stimulated CD14+ monocytes.

	Gene	Function	p-value	p adj-value
♂	*HTRA1*	Inhibits TGFb singnaling, indirectly	0.0019	0.99996
	PPP1R11	Inhibits protein phoyphatases, ubiquititation of TLR2	0.0035	0.99996
	IL36RN	Inhibits NFkB activation, indirectly	0.0175	0.99996
	DDAH1	Inhibits NO synthase activity, indirectly	0.0196	0.99996
♀	*EXOSC6*	mRNA degradation	8.13E-09	1.57E-05
	RGS1	Attenuation of the signaling activity of G-proteins	0.0004	0.45813
	CST7	Glycosylated protease with a putative role in immune regulation	0.0036	0.99996
	ZBTB18	Transcriptional repressor	0.0045	0.99996
	SIRPB1	Involved in negative regulation of receptor tyrosine kinase-coupled signaling processes	0.0048	0.99996
	DAPK1	Related to apoptosis	0.0058	0.99996
	MAGEH1	Related to apoptosis, cell cycle arrest, growth inhibition, cell differentiation	0.0059	0.99996
	PPP1R16B	Regulator of protein phosphatase 1, regulation of the PI3K/AKT signaling pathway	0.0069	0.99996
	PHLDA3	Inhibition of Akt1	0.0078	0.99996
	RASA3	Negative regulator of the Ras signaling pathway	0.0094	0.99996
