# Supplementary material for: Androgens predispose males to monocyte-mediated immunopathology by inducing the expression of leukocyte recruitment factor CXCL1
Source: Nat Commun. 2020 Jul 10;11:3459. doi: 10.1038/s41467-020-17260-y (PMC7351718; doi:10.1038/s41467-020-17260-y)
Supplement: Supplementary file 3 — Reporting Summary [file 41467_2020_17260_MOESM3_ESM.pdf]

nature research

Corresponding author(s): Prof. Dr. Hanna Lotter

Last updated by author(s): Mar 27, 2020

Reporting Summary

Nature Research wishes to improve the reproducibility of the work that we publish. This form provides structure for consistency and transparency in reporting. For further information on Nature Research policies, seeAuthors & Referees and theEditorial Policy Checklist .

Statistics

For all statistical analyses, confirm that the following items are present in the figure legend, table legend, main text, or Methods section.

n/a	Confirmed	
☐	☒	The exact sample size (n) for each experimental group/condition, given as a discrete number and unit of measurement
☐	☒	A statement on whether measurements were taken from distinct samples or whether the same sample was measured repeatedly
☐	☒	The statistical test(s) used AND whether they are one- or two-sided<br>*Only common tests should be described solely by name; describe more complex techniques in the Methods section.*
☐	☒	A description of all covariates tested
☐	☒	A description of any assumptions or corrections, such as tests of normality and adjustment for multiple comparisons
☒	☐	A full description of the statistical parameters including central tendency (e.g. means) or other basic estimates (e.g. regression coefficient) AND variation (e.g. standard deviation) or associated estimates of uncertainty (e.g. confidence intervals)
☒	☐	For null hypothesis testing, the test statistic (e.g. F, t, r) with confidence intervals, effect sizes, degrees of freedom and P value noted<br>*Give P values as exact values whenever suitable.*
☒	☐	For Bayesian analysis, information on the choice of priors and Markov chain Monte Carlo settings
☒	☐	For hierarchical and complex designs, identification of the appropriate level for tests and full reporting of outcomes
☒	☐	Estimates of effect sizes (e.g. Cohen's d, Pearson's r), indicating how they were calculated

Our web collection on statistics for biologists contains articles on many of the points above.

Software and code

Policy information about availability of computer code

Data collection	The BD LSRFortessa was controlled by the BD FACSDiva V8 software. The MRI screening was controlled by NUMARIS/4 syngo MR B15. The data collection of the RT-PCR was perfomed by the Roche Lightcycler® 96 software. The measurement of optical densities of ELISA experiments was performed with the MRX® and cotrolled by the RevelationTM software. RNAseq data from RNA samples was performed and collected by BGI Genomics.
Data analysis	Abscess volumes were analyzed using either the Osirix DICOM Viewer or ImageJ. Flow cytometry analysis was performed by FlowJo V10. HSNE analysis was performed by cytosplore Version 2.2.1.. LEGENDplexTM analysis was conducted with the LEGENDplex data analysis software V8.0. Analysis of RNAseq data was conducted using HISAT2 and featureCounts including the subreads package. Further transcriptomic analysis were performed using the Deseq2 software, GOrilla and Panther GO-slim algorithm as well as with the heatmapper.ca software. Statistical analysis were perfomed with Graphpad Prism V8.1.2.

For manuscripts utilizing custom algorithms or software that are central to the research but not yet described in published literature, software must be made available to editors/reviewers. We strongly encourage code deposition in a community repository (e.g. GitHub). See the Nature Research guidelines for submitting code & software for further information.

Data

Policy information about availability of data

All manuscripts must include a data availability statement. This statement should provide the following information, where applicable:

- Accession codes, unique identifiers, or web links for publicly available datasets
- A list of figures that have associated raw data
- A description of any restrictions on data availability

The data that support the findings of this study are available from the corresponding author upon reasonable request.

Field-specific reporting

Please select the one below that is the best fit for your research. If you are not sure, read the appropriate sections before making your selection.

- [x] Life sciences
- [ ] Behavioural & social sciences
- [ ] Ecological, evolutionary & environmental sciences

For a reference copy of the document with all sections, see nature.com/documents/nr-reporting-summary-flat.pdf

Life sciences study design

All studies must disclose on these points even when the disclosure is negative.

Sample size	No statistical method was used to determine sample size. Sample size were chosen based on previous experience. Especially animal experiments needed to fit the 3R rule, but enough to fit profound statistical calculations. Human data were collected based on availability.
Data exclusions	No data were excluded from the analysis, exept for RNAseq analysis. The purity of the isolated CD14 positive monocytes had to reach at least 90 %.
Replication	Animal experiments were done in duplicate. Human data were done once. All other experiments were conducted in triplicate.<br>All attempts of replication were successful and gave similar results.
Randomization	Male and female mice couldn't sit together in randomized groups, but animals were assigned randomly to experimental and control groups.
Blinding	The investigators were not blinded during data collection, but the FlowJo analysis were conducted blinded.

Reporting for specific materials, systems and methods

We require information from authors about some types of materials, experimental systems and methods used in many studies. Here, indicate whether each material, system or method listed is relevant to your study. If you are not sure if a list item applies to your research, read the appropriate section before selecting a response.

Materials & experimental systems

n/a	Involved in the study	
[ ]	[x]	Antibodies
[x]	[ ]	Eukaryotic cell lines
[x]	[ ]	Palaeontology
[ ]	[x]	Animals and other organisms
[ ]	[x]	Human research participants
[x]	[ ]	Clinical data

Methods

n/a	Involved in the study	
[x]	[ ]	ChIP-seq
[ ]	[x]	Flow cytometry
[ ]	[x]	MRI-based neuroimaging

Antibodies

Antibodies used

Antibodies against murine epitopes:
BioLegend; CD11b; BV510; clone: M1/70; lot: B265262
BioLegend; CD86; AF700; clone: GL-1; lot: B268638
BioLegend; CCR2; PE-Cy7; clone: SA203G11; lot: B241310
BioLegend; Ly6C; FITC; clone: HK1-4; lot: B247728
BioLegend; Ly6C; PE; clone: HK1-4; lot: B148724
BioLegend; Ly6C; PerCP/C5.5; clone: HK1-4; lot: E10161-1631
BioLegend; Ly6G; APC-Cy7; clone: 1A8; lot: B264760
BioLegend; Ly6G; AF700; clone: 1A8; lot: B166379
BioLegend; Ly6G; BV785; clone: 1A8; lot: B262919
BioLegend; CX3CR1; PerCP/5.5; clone: SA011F11; lot: B250966
BioLegend; TNFalpha; BV421; clone: MP6-XT22; lot: B205119
BioLegend; CCL2; PE; clone: 2H5; lot: B273678
R&D; CXCL1; Alexa Fluor 647; clone: 1174A; lot: 1518157

Antibodies against human epitopes:
BioLegend; CD14; Alexa Fluor 700; clone: M5E2; lot: B239383
BioLegend; CD14; PerCP/Cy5.5; clone: M5E2; lot: B239204
BioLegend; CD16; PerCP; clone: 3G8; lot: B235385
BioLegend; CCR2; Allophycocyanine; clone: K036C2; lot: B183753

	BioLegend; CCR5; Alexa Fluor 488; clone: J418F1; lot: B240234<br>BioLegend; CX3CR1; PE/Cy7; clone: 2A9-1; lot: B246272<br>R&D; TNFRI; clone: 16803; lot: IP0917071<br>R&D; TNFRII; clone: 22210; lot: AYF0417041<br>R&D; TNFalpha, clone1825; lot: HM3717081<br>Becton Dickinson; HLA-DR; BUV395; clone: G46-6; lot: 8205839<br>Abcam; Androgen Receptor; Alexa Fluor 488; clone: ER179(2); lot: GR238755<br>Abcam; Rabbit IgG; Alexa Fluor 488; clone: EPR25A; lot: GR274056<br><br>Secondary antibody:<br>ThermoFisher Scientific anti-mouse IgG (H+L) Alexa Fluor 594 clone: #A-11005; lot: 1697164<br>R&D isotype control clone: 11711; lot: IX2410121
Validation	All antibodies were titrated with the cells of interest and checked with the help of the appropriate isotype control. The control stainings for Androgen Receptor, TNFRI and TNFRII are shown in the supplementary information of the manuscript.<br>The concentration for the neutralizating antibodies against TNF, TNFRI and TNFRII from R&D were derived from the manufactor's guidelines and cited references.

Animals and other organisms

Policy information about studies involving animals; ARRIVE guidelines recommended for reporting animal research

Laboratory animals	C57BL/6J male and female mice were used for this study. At the time of the experiments the mice were 8 to 12 weeks old. More details on the mice used in this study have been included in the Methods section of this manuscript.
Wild animals	The study did not involve wild animals.
Field-collected samples	The study did not provide samples collected from the field.
Ethics oversight	Animal experiments were performed under the agreement of the German animal protection law and were reviewed by the federal health authorities of the State of Hamburg (Behörde für Gesundheit und Verbraucherschutz) in accordance of the ARRIVE guidelines and registered under the permission number 51/17 and 01/15.

Note that full information on the approval of the study protocol must also be provided in the manuscript.

Human research participants

Policy information about studies involving human research participants

Population characteristics	The study included only healthy male and female participants, which were between 25 and 49 years old.
Recruitment	Information of human blood donors were restricted to health status, sex and age for both study protocols.
Ethics oversight	Both study protocols (PV5252 and PV5245) were approved by the committee of the medical association Hamburg.

Note that full information on the approval of the study protocol must also be provided in the manuscript.

Flow Cytometry

Plots

Confirm that:

- [ ] The axis labels state the marker and fluorochrome used (e.g. CD4-FITC).
- [ ] The axis scales are clearly visible. Include numbers along axes only for bottom left plot of group (a 'group' is an analysis of identical markers).
- [x] All plots are contour plots with outliers or pseudocolor plots.
- [ ] A numerical value for number of cells or percentage (with statistics) is provided.

Methodology

Sample preparation	The detailed sample preparation in listed is the method section of the manuscript.
Instrument	BD LSRFortessa
Software	BD FACSDiva V8

Cell population abundance	A fraction of the purified monocytes via anti-Human CD14 Magnetic Particles Kit (Becton Dickinson) were stained using an antiCD14 PerCP/Cy5.5 antibody. The purity of the cell fraction was analyzed by flow cytometry. Samples with a purity under 90 % were excluded for further RNAseq analysis. The normal frequency of CD14 positive monocytes out of PBMCs is about 10 %.
Gating strategy	The gating strategy of murine monocytes is expemplarly shown in the manuscript as well as the gating strategy for human monocytes, which was based on the strategy by Heimbeck et al., 2010.

☐ Tick this box to confirm that a figure exemplifying the gating strategy is provided in the Supplementary Information.

Magnetic resonance imaging

Experimental design

Design type	no neuroimaging, but resting state. The volume of the abscess were documented by MRI
Design specifications	the abscess size was measured 3 times from each individual
Behavioral performance measures	n/a

Acquisition

Imaging type(s)	Structural measurement
Field strength	7 Tesla
Sequence & imaging parameters	T2-weighted turbo spin echo (T2wTSE) sequence; field of view 32 mm; matrix size 256 x 256; slice thickness 0,8 mm; orientation transversal; TE 51 msec / TR 1460 msec (the echotrainlength was 14) / flipangle 180 degrees
Area of acquisition	abdomen, the organ of interest was the liver and was determined with a low resolution localizer

Diffusion MRI ☐ Used ☒ Not used

Preprocessing

Preprocessing software	NUMARIS/4 syngo MR B15
Normalization	not normalized, because ROI-based volumemetric measurements were performed
Normalization template	n/a
Noise and artifact removal	no additional noise or artefact removal
Volume censoring	n/a

Statistical modeling & inference

Model type and settings	To reduce animal numbers, the abscess development (volumetric analysis) of the mice was documented 3 times in a week in each mouse per MRI.
Effect(s) tested	ROI-based volumemetric measurements

Specify type of analysis: ☐ Whole brain ☒ ROI-based ☐ Both

Anatomical location(s): abdomen, the organ of interest was the liver and was determined with a low resolution localizer. The region of interest was manually determined by the user.

Statistic type for inference (See Eklund et al. 2016)	n/a
Correction	n/a

Models & analysis

n/a	Involved in the study
☒	☐ Functional and/or effective connectivity
☒	☐ Graph analysis
☒	☐ Multivariate modeling or predictive analysis
